# Occludin deficiency is associated with developmental impairment, locomotor dysfunction, and social deficiencies

**DOI:** 10.1080/21688370.2026.2647450

**Published:** 2026-03-24

**Authors:** Silvia Torices, Nikolai Fattakhov, Oandy Naranjo, Destiny Daniels, Sierra Simon, Leah Daire, Luisa Mendoza, Marta Kopanska, Michal Toborek

**Affiliations:** a Department of Biochemistry and Molecular Biology, University of Miami Miller School of Medicine, Miami, FL, USA; b Department of Medical Psychology, Faculty of Medicine, Collegium Medicum, University of Rzeszow, Rzeszow, Poland; c Institute of Physiotherapy and Health Sciences, The Jerzy Kukuczka Academy of Physical Education, Katowice, Poland

**Keywords:** Autism spectrum disorder, blood-brain barrier, motor dysfunction, neurodevelopmental disorders, occludin

## Abstract

Neurodevelopmental and psychiatric disorders, such as attention deficit hyperactivity disorder or autism spectrum disorder, have been intricately linked to structural impairments and compromised permeability of the blood-brain barrier (BBB). Occludin (ocln) is a tight junction (TJ) protein known for its role in maintaining BBB integrity. In the present manuscript, we report unexpected connection between ocln deficiency and behavioral alterations resembling neurodevelopmental and psychiatric disorders. Adolescent, occludin-deficient (ocln^−/−^) mice displayed an abnormal growth and neurological phenotype along with deterioration of motor functions as compared to wild-type controls. Moreover, adult ocln^−/−^ mice exhibited increased hyperactivity, social difficulties, and attention deficits compared to control mice. Among the brain structures, the hippocampus appeared to be most susceptible to ocln deficiency as it exhibited higher expression levels of ocln than both the frontal cortex and striatum. Overall, the observed behavioral alteration patterns in ocln^−/−^ mice indicated pronounced neurological motor dysfunction and behavioral changes that parallel those seen in attention deficit hyperactivity disorder and autism spectrum disorder. These findings are important for better understanding the role of ocln in neurodevelopmental and psychiatric disorders.

## Introduction

Neurodevelopmental disorders (NDDs) involve a heterogeneous group of disorders characterized by clinical and genetic variabilities that affect the function and development of the brain.^1^ Individuals with NDDs are more susceptible to developing mental health problems, such as autism spectrum disorder and attention‐deficit hyperactivity disorder.^1,2^ Autism spectrum disorder is a common heterogeneous disorder marked by challenges in social communication, interaction, and repetitive behaviors.^3^ Moreover, people with autism often suffer from anxiety, deficits in learning, hyperactivity and aggressive behaviors.^4,5^ The disruption and heightened permeability of the blood-brain barrier (BBB) have been suggested to be a prevalent factor across these disorders.^6^

As the intermediary interface between the brain and peripheral blood, the integrity of the BBB regulates brain homeostasis. Tight junctions (TJs) play a pivotal role in restricting the passage of molecules across the brain endothelium, ensuring barrier functionality.^7^ TJ damage has been linked to various neurological disorders that impact the central nervous system (CNS).^6,8,9^ Emerging evidence implicates TJ proteins, especially claudin-5 in several neurological and psychiatric diseases, including epilepsy,^10^ bipolar disorder,^11^ and autism spectrum disorder.^12^ It was demonstrated that the *CLDN5* gene, which encodes claudin-5, harbored heterozygous missense variants in patients exhibiting developmental delay, seizures, microcephaly, and a recognizable pattern of pontine atrophy and brain calcifications.^13^ Furthermore, higher levels of methylation in CLDN5 were associated with cognitive decline, implicating the important role of this BBB protein in the maintenance of cognitive trajectory with aging.^14^

In contrast to claudin-5, the role of occludin (ocln) deficiency in the development of psychiatric and neurodevelopmental-type disorders remains elusive. Ocln is a 65-kDa integral transmembrane protein,^15^ which is expressed in variety of cell types throughout the body. Aside from its role in regulation of barrier integrity, ocln can also be present in the cytoplasm ^16–18^ and act as a multifunctional protein influenced by its phosphorylation status.^19^ In fact, ocln was demonstrated to act as an NADH oxidase, influencing cellular metabolism by modulating the AMPK protein kinase activity.^18,20^ Increased serum levels of ocln were observed in children with autism spectrum disorder,^12^ implicating ocln in the onset of this disease. Moreover, reduced expression of ocln has been linked to compromised intestinal and BBB permeability and neurodevelopmental impairment in preterm infants.^21^

In this study, we report unexpected findings related to the developmental impairment and behavioral abnormalities of ocln-deficient mice, which encompass hyperactivity, social deficits, and memory impairments, resembling the characteristics of attention deficit hyperactivity disorder and autism spectrum disorder.

## Materials and methods

### Mice

Global ocln knockout (KO) mice were use for this study. Ocln^±^ mouse embryos were generously provided by the Kumamoto University (Center for Animal Resources and Development, Kumamoto, Japan). Genotyping was performed on ear punch tissue. All procedures were approved by the University of Miami Institutional Animal Care and Use Committee (IACUC) and were consistent with the National Institutes of Health (NIH) guidelines. Experiments were conducted in both sexes. Age- and sex-matched ocln^−/−^, ocln^±^, and ocln^+/+^ mice were randomly assigned to the respective experimental groups. Mice which exhibited a sick behavior were excluded from the study. All experiments conformed to the ARRIVE guidelines for *in vivo* research. Number of mice per group was established based on power analysis assuming a 20–30% difference in biological variables to be necessary to observe statistically significant differences.

### Neurologic and motor function assessment

Differences in the body weight, length, and neurodeficit score were evaluated at the ages of 3, 6, 9, and 12 weeks. Neurologic motor function was assessed by performing the composite neuroscore. Animals were scored from 0, considered severely impaired to 4 considered normal in the following parameters^1^: tail suspension,^2^ walking test, and ^3^ resistance to lateral right and left pulsion as previously described.^22^ The maximum score per animal, considered normal, was 12. The researchers performing the studies were blind to the study group, which were revealed only at the end of the study.

### Footprint analysis

The assessment of gait was performed using footprint analysis in adolescent 3 week-old-mice. Briefly, after covering the feet with nontoxic ink (red for ocln^−/−^, green for ocln^±^, and blue for ocln^+/+^ mice), mice were allowed to walk over white paper in a 50-cm-long, 8-cm high, and 10-cm-wide tunnel. The print patterns were analyzed for the following parameters: the average distance between alternate steps (stance length), the average distance between left and right hind (sway length), and the average distance between each stride (stride length) in a forward movement. All measures were represented in centimeters (cm).

### Behavioral assessments

Before each experiment, mice were allowed to habituate to the experimental room for 30 minutes before the test. White noise was used during habituation and the experimental test at 60 Db. All experiments were blinded in the manner conducted. All experiments were performed in adult mice at the age of 12-week-old to address the questions if early developmental changes can influence adult behavioral responses.

#### Open field

Mice were placed in the center of the apparatus (Stoelting Co., Wood Dale, USA, Cat# 60,101), which consisted of a square area (40 cm × 40 cm) surrounded by gray plastic walls. Mice were allowed to freely move for 10 minutes, and the activity of the following parameters was monitored, recorded, and analyzed using the activity monitoring tracking software, ANY-mazeTM Video Tracking System (Stoelting Co.,): total distance traveled, average speed, number of entries, and time spent in the peripheral and central zones.

#### Social interaction and social recognition

Sociability Chamber Box from Noldus (Leesburg, VA, USA) was employed. The box was formed by three chambers connected by doors. The same-age and sex, never-before-met stimulus (stranger 1) mouse was placed in one of the chambers under one crate, and another empty cup was placed in the other chamber of the box. Mice in the study were allowed to explore both chambers for 10 minutes. Using the ANY-mazeTM Video Tracking System, we tested social interactions by measuring the time that the mice in the study spent interacting (e.g., touching, sniffing) with the never-before-met stimulus mice. Social recognition was evaluated by placing in one chamber of the box a “familiar” mouse used to measure social interaction and in the other chamber a ‘novel’ never-before-met stimulus mouse (stranger 2). The mice were then allowed to explore the “stranger 1” and “stranger 2” mice for 10 minutes and social recognition was evaluated by measuring the time that the mice in the study spent interacting with each stranger mouse.

#### Novel object recognition

The Novel Object Recognition test was assessed using the sociability chamber box previously described. By conducting both the NOR and the social interaction tests in the same apparatus, we sought to reduce context-dependent anxiety and improve within-subject comparability. All objects were thoroughly cleaned with 70% ethanol between trials to eliminate residual odor cues, and object positions were counterbalanced to avoid side preference biases. Mice were individually habituated 24 hours before the testing by allowing them to freely explore an object for 10 minutes. The next day, a mouse was placed in the box with the same object from the previous day for 5 minutes. The mouse was then returned to the house cage for 10 minutes. The test was performed by placing the mouse in the chamber again for 10 minutes with the same familiar object used before and a new object. Items used between tests were cleaned with ethanol to eliminate residual odors. Using the tracking software ANY-mazeTM Video Tracking System, we measured the time that the mouse of the study spent interacting with each object. The percentage of exploration time was calculated to represent the proportion of time the test mouse spent investigating the novel object relative to total object exploration, using the following formula: (Tnovel)/(Tnovel + Tfamiliar) × 100 indicate the time spent exploring the novel and familiar objects, respectively. To further assess recognition memory, a Discrimination Index (DI) was calculated to evaluate the animal’s relative preference for the novel object while accounting for total exploration time. The DI was computed using the formula DI = (Tnovel -Tfamiliar)/(Tnovel +Tfamiliar) where Tnovel is the time exploring the novel object and Tfamiliar is the time exploring the familiar object.

## Western blotting

The frontal cortex, hippocampus, and striatum were lysed with 200 µl of RIPA buffer containing protease inhibitors (Santa Cruz Biotechnology, Dallas, TX, USA, Cat# sc-24948a). Protein levels were quantified using the BCA Protein Assay Kit (Thermo Fisher Scientific, Cat# 23,223). Samples were electrotransferred in 4–20% Mini-PROTEAN TGX Stain-Free Protein Gel and PVDF membrane Trans-Blot Turbo Transfer System (Bio-Rad Laboratories, Hercules, CA, USA, Cat# 170–4159). Nonspecific binding was blocked with bovine serum albumin (BSA) at 5% in TBS-0.05% Tween20 for 1 hour; then, the membranes were incubated overnight (1:1000 in 5% BSA-TBS) with the following primary antibodies: anti-occludin (Thermo Fisher Scientific, Cat# 71‐1500), anti-ZO (Thermo Fisher Scientific, Cat# 33–9100), anti-Pcam-1 (Cell Signaling, Cat# 3528S), anti-claudin5 (Cell Signaling, Cat# 49,564). Next day, blots were washed with TBS-0.05% Tween20 three times for 5 minutes and incubated for 1 hour at 1:20000 in 5% BSA-TBS with the following secondary antibodies from LI-COR Biosciences (Lincoln, NE, USA) Cat# 926–32210, Cat# 926–68070, Cat# 926–32211, and Cat# 926–68071. Anti-β-actin antibody was used as a housekeeping (Thermo Fisher Scientific, Cat#MA5-15739). Blots were analyzed using the Licor CLX imaging system and the Image Studio 4.0 software (LI-COR).

## Measurement of BBB permeability

Mice were injected intravenously through the tail vein with 200 µL of a 10% sodium fluorescein (NaF) (Sigma-Aldrich, Cat# F6377-100 G). After 20 minutes, the blood was collected by heart puncture, and the brains were harvested after perfusing the body with saline. The frontal cortex, striatum, and hippocampus were extracted from each hemisphere and homogenized in PBS. Protein levels were quantified by the BCA Protein Assay Kit (Thermo Fisher Scientific, Cat# 23,223). Samples were incubated overnight with 100% Trichloroacetic acid (TCA) (Sigma, Cat# T6399) to extract the NaF from the tissues and then neutralized with 0.05 M Sodium Tetraborate buffer. The following day, supernatant absorbance was measured at 480 nm excitation and 538 nm emission. NaF extravasation into the brain was normalized to protein levels and NaF levels in plasma.

## Statistical analysis

Statistical analyses were performed using GraphPad Prism Software (La Jolla, CA, USA). Statistical significance was determined by one- or two-way ANOVA followed by Tukey’s multiple comparisons test, or Unpaired *t* test with or without Welch’s test correction and established at *p* < 0.05 (****p < 0.0001, ***p = 0.0002, **p = 0.003, *p < 0.0449). For normality and homogeneity of variance, we conducted the Shapiro Wilk test and Kolmogorov-Smirnov and Brown-Forsythe test respectively. Statistical tests employed are listed in Figure Legends. In addition, comprehensive statistical information of all reported results is presented in Supplementary Table 1.

## Results

### Growth and neuroscore abnormalities of ocln deficient mice

**Figure 1.**
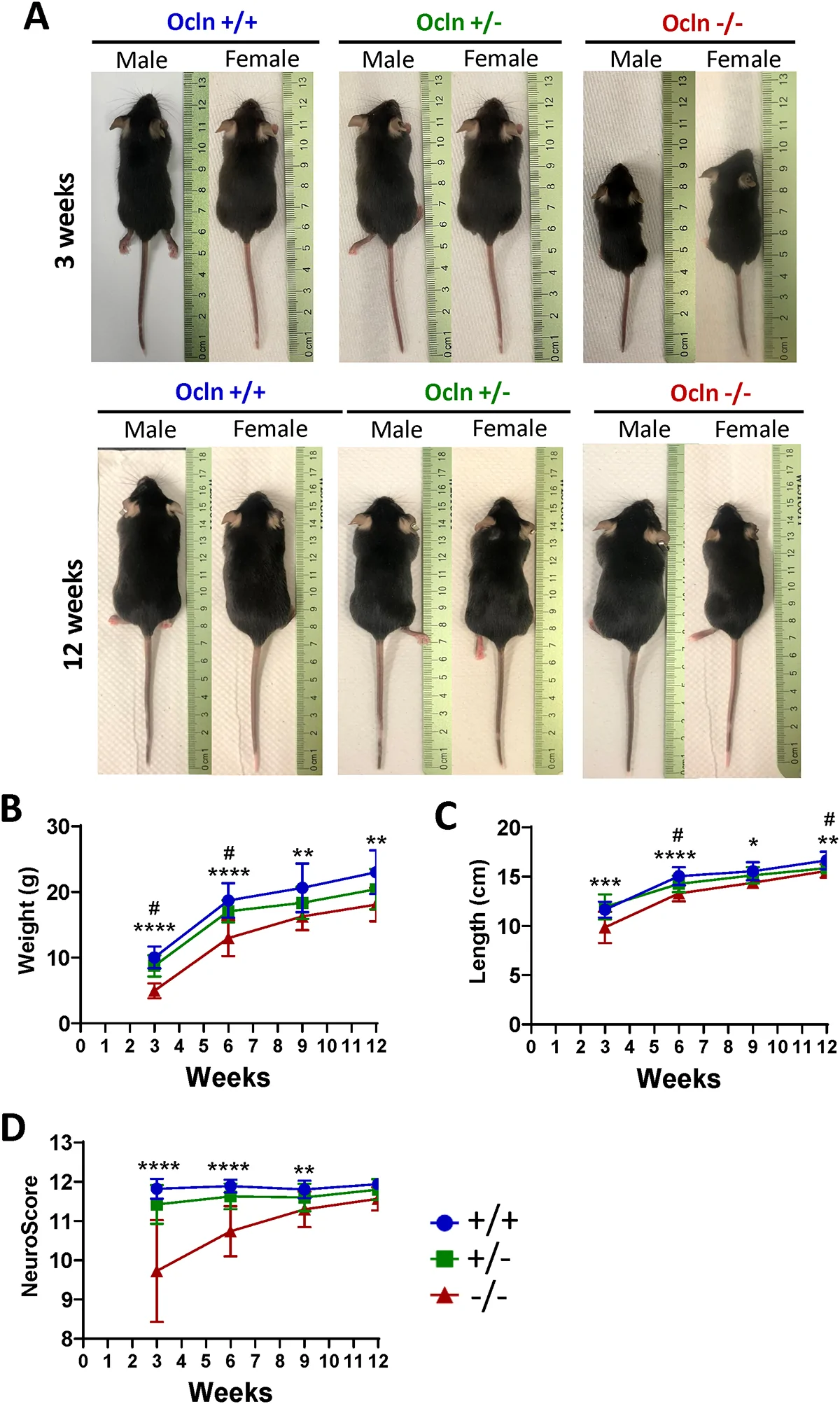
Ocln deficient mice exhibit growth impairment. (A) Representative images of 3-week-old and 12-week-old ocln^+/+^, ocln^±^, and ocln^−/−^ mice. (B) body weight, (C) length, and (D) neuroscore of 3, 6, 9, and 12-week-old ocln^+/+^, ocln^±^, and ocln^−/−^ mice. Individual data points are marked in blue (ocln^+/+^), green (ocln^±^), and red (ocln^−/−^) dots. Graphs indicate the mean ± sd from three independent experiments. * ocln^+/+^ vs ocln^−/−^ groups at ****p < 0.0001, ***p = 0.0002, **p = 0.003, *p < 0.0449; ^#^ ocln^±^ vs ocln^−/−^ groups at ^####^*p* < 0.0001, ^#^*p* < 0.00449; ^π^ ocln^+/+^ vs ocln^±^ groups at ^ππ^*p* = 0.003, ^π^*p* < 0.0449; *n* = 10–18 animals per group; two-way ANOVA. Data applies to both sexes.

**Figure 2.**
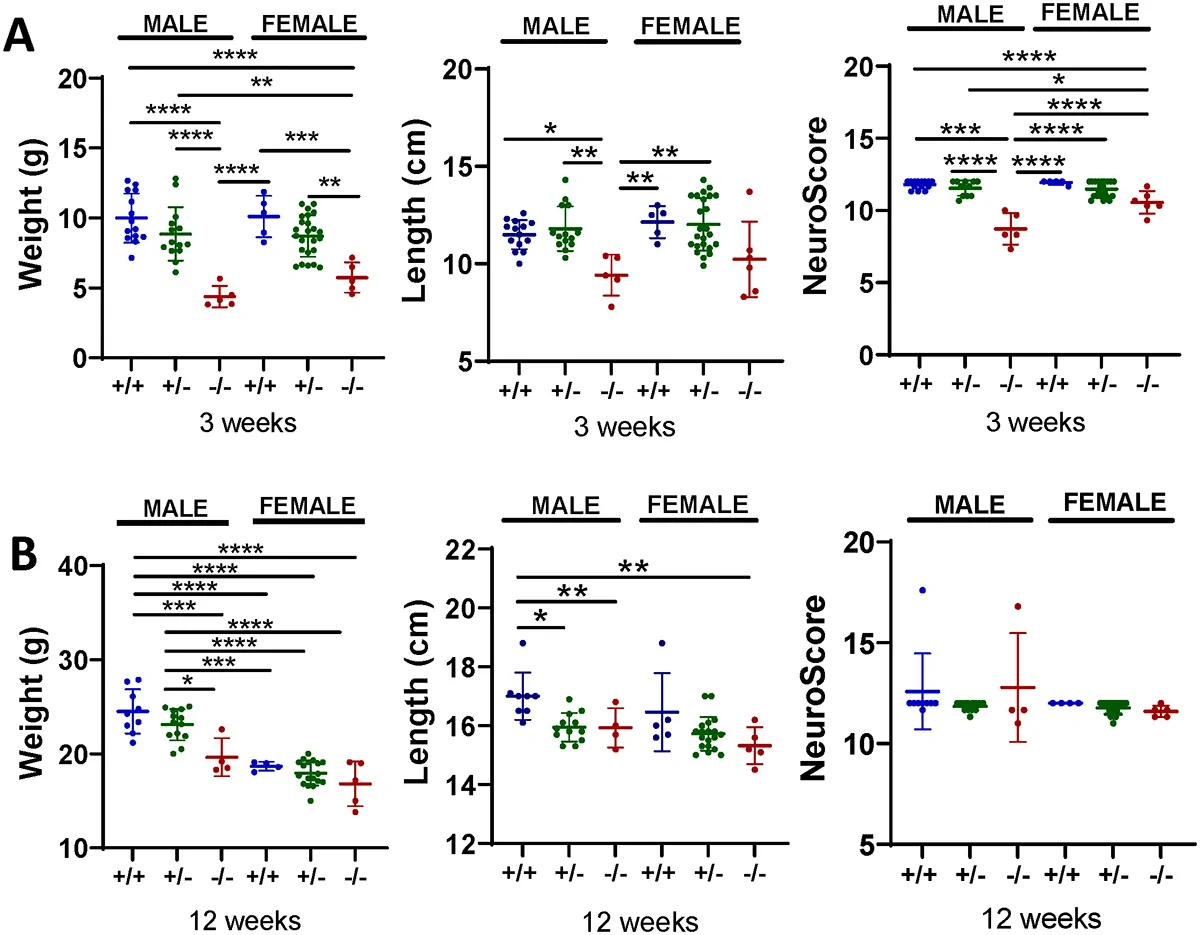
Impact of gender on growth and neuroscore impairment of ocln-deficient mice. (A) Body weight, length, and neuroscore by gender of 3-week-old ocln^+/+^, ocln^±^, and ocln^−/−^ mice. (B) Body weight, length, and neuroscore by gender of 12-week-old ocln^+/+^, ocln^±^, and ocln^−/−^ mice. Individual data points are marked in blue (ocln^+/+^), green (ocln^±^), and red (ocln^−/−^) dots. Graphs indicate the mean ± SD from three independent experiments. ****p < 0.0001, ***p = 0.0002, **p = 0.003, *p < 0.0449, *n* = 4–14 animals per group; two-way ANOVA. Data applies to separate sexes.

The body weight of ocln^−/−^, ocln^±^, and ocln^+/+^ mice was monitored between the ages of 3 and 12 weeks both in males and females. Adolescent ocln^−/−^ mice presented a significantly lower weight and shorter body length at the ages of 3 and 6 weeks when compared to ocln^±^ or ocln^+/+^ mice (Figure 1A upper panel, B, C). Interestingly, these differences were largely attenuated by the ages of 9 and 12 weeks (Figure 1A **lower panel, B, C)**. Additionally, we assessed motor functions by measuring the composite neuroscore according to previously established methods.^22^ Ocln^−/−^ mice exhibited diminished motor skills during early adolescence as compared to ocln^±^ or ocln^+/+^ mice. Notably, these deficits diminished in mice aged 9 weeks and largely disappeared in mice aged 12 weeks (Figure 1D). Growth and neuroscore deficits were more pronounced in male mice compared to female mice at 3 weeks of age (Figure 2A); however, these differences diminished by 12 weeks of age (Figure 2B).

### Impaired locomotor functions and hyperactivity in ocln deficient mice

**Figure 3.**
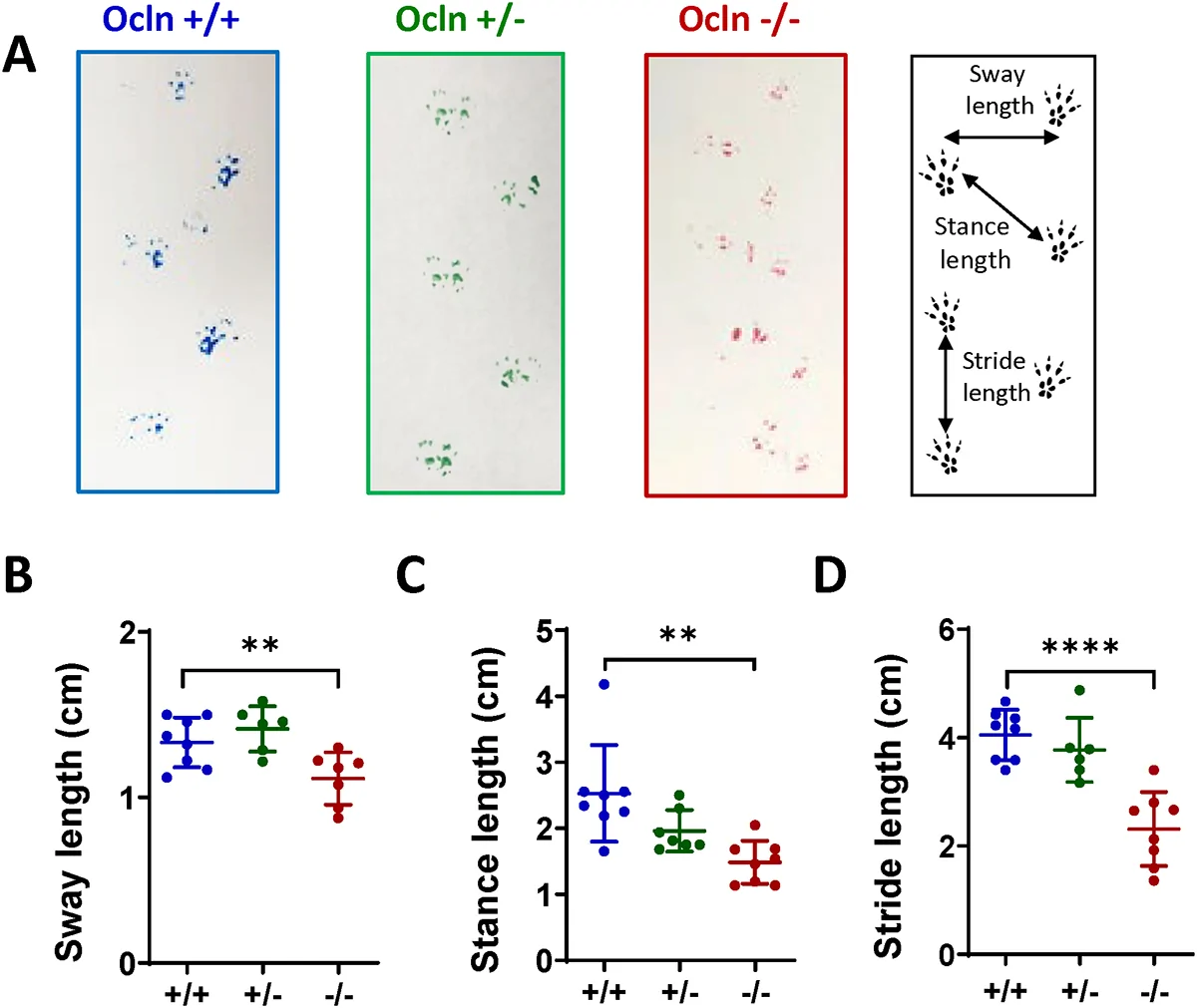
Ocln-deficient mice display locomotor impairment. (A) Representative footprint images of 3-week-old ocln^+/+^, ocln^±^, and ocln^−/−^ mice and a diagram illustrating the footprint analysis. Quantification of the sway (B), stance (C), and stride (D). Individual data points are marked in blue (ocln^+/+^), green (ocln^±^), and red (ocln^−/−^) dots. Graphs indicate the mean ± sd from three independent experiments. ****p < 0.0001, ***p = 0.0002, **p = 0.003, *p < 0.0449, *n* = 6–8 animals per group; one-way ANOVA. Data applies to both sexes.

To confirm the role of ocln deficiency in aberrant locomotor functions, footprint analysis was utilized on ocln^−/−^, ocln^±^, and ocln^+/+^ mice at 3 weeks of age. Figures 3A-D demonstrate that ocln^−/−^ mice exhibited significantly shorter sway, stance, and stride lengths in this early adolescence stage, when compared with ocln^±^ or ocln^+/+^ mice.

**Figure 4.**
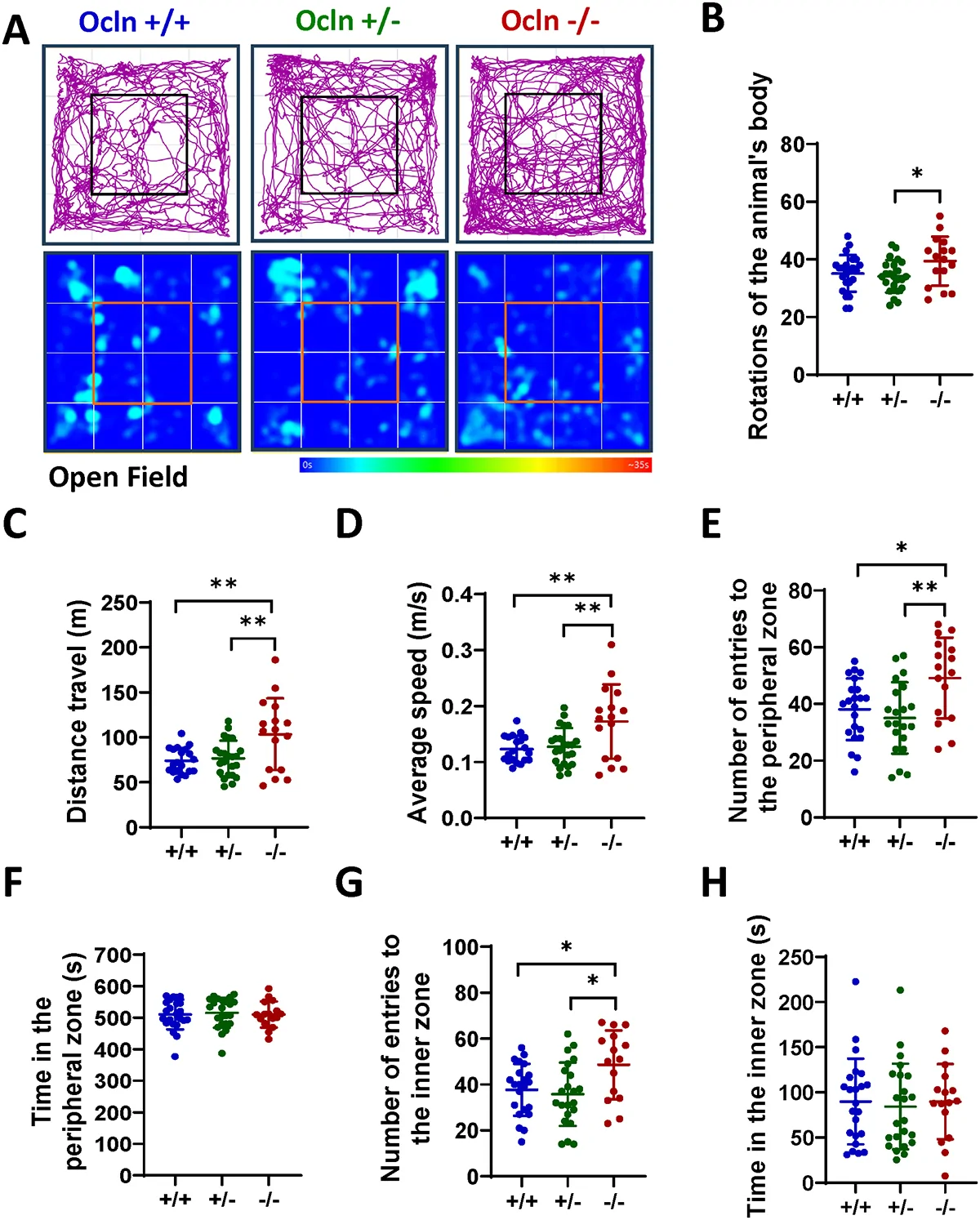
Hyperactivity in ocln-deficient mice. Open field test was recorded and analyzed using activity monitoring tracking software, ANY-mazeTM Video tracking System. (A) Representative track pathway and heat map images of ocln^+/+^, ocln^±^, and ocln^−/−^ mice during the test. (B) body rotations, (C) total distance traveled, (D) average speed, (E) number of entries to the peripheral zone, (F) time in the peripheral zone, (G) number of entries to the inner zone, and (H) time in the inner zone. Individual data points are marked in blue (ocln^+/+^), green (ocln^±^), and red (ocln^−/−^) dots. Graphs indicate the mean ± sd from three independent experiments. **p = 0.003, *p < 0.0449, *n* = 16–21 animals per group; one-way ANOVA. Data applies to both sexes.

We next investigated spontaneous motor activity and emotional responses in a novel environment. Specifically, the open field test was performed to assess alterations in motor activity, exploratory behaviors, and anxiety levels. The tests were performed on 12-week-old adult mice to address the questions if early adolescent changes can influence these parameters in adult mice. The numbers of rotations of the animal´s body, the total distance traveled, and the average speed were increased in ocln^−/−^ mice as compared with wild-type controls (Figures 4A-D). Ocln^−/−^ mice were also characterized by increased number of entries to both the peripheral and the inner zone as compared to controls. In contrast, the time spent by mice in the peripheral and the inner zones was the same between the studied groups (Figures 4E-H). These results, coupled with an increased average speed and distance traveled by ocln^−/−^ mice, suggest an association between ocln deficiency and hyperactive behavior. No changes were found between genders when analyzing these parameters **(Supplementary Figures S1A-D)**.

### Aberrant social interaction behavior in ocln deficient mice

**Figure 5.**
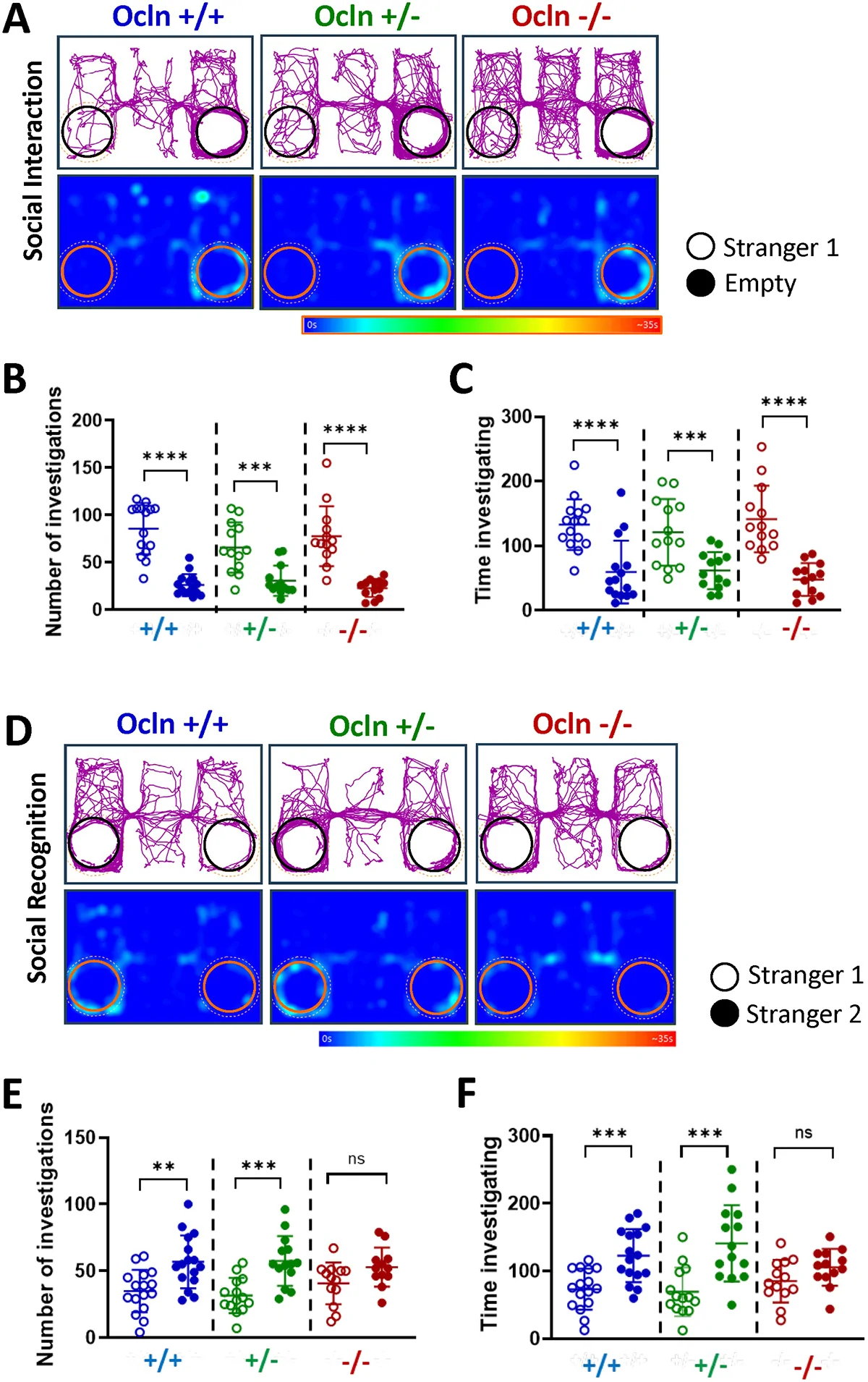
Ocln deficient mice exhibit aberrant social interactions. Social novelty and social interaction tests were recorded and analyzed using activity monitoring tracking software, ANY-mazeTM Video tracking System. (A) Representative track pathway and heat map images of the social novelty test of ocln^+/+^, ocln^±^, and ocln^−/−^ mice. (B) number of investigations and (C) time spent investigating a stranger mouse and an empty box. (D) Representative track pathway and heat map images of social interaction test of ocln^+/+^, ocln^±^, and ocln^−/−^ mice. (E) the number of investigations and (F) time spent investigating a stranger 1 and (F) time spent investigating a stranger 2 mouse. Individual data points are marked by blue (ocln^+/+^), green (ocln^±^), and red (ocln^−/−^) dots and circles. Graphs indicate the mean ± sd from three independent experiments. ****p < 0.0001, ***p = 0.0002, **p = 0.003, *n* = 14–21 animals per group; unpair t-test. Data applies to both sexes.

To evaluate social behavior, we conducted social interaction and social recognition tests, focusing on cognitive abilities related to interactions with other animals and distinguishing between novel and familiar mice. In the initial part of the social interaction test, an unfamiliar mouse (stranger 1) was placed under a crate on one side of the chamber and an empty cup was placed in the other chamber. Mice in all studied groups exhibited strong preferences toward number of interactions and time spent interacting with an unfamiliar mouse (stranger 1) as compared to the empty chamber of the box. No differences between groups were noted in this part of the test (Figures 5A-C). In the second part of the social recognition test, the first unfamiliar mouse (stranger 1) remained under the same crate and served now as a familiar mouse. A “novel” never-before-met stimulus mouse (stranger 2) was introduced under another crate in the other chamber of the box. In this experimental setting, ocln^+/+^ and ocln^±^ mice exhibited a notable increase in the time spent and number of nose contacts around the chamber containing the unfamiliar mouse (stranger 2). Conversely, ocln^−/−^ mice did not display any discernible preferences. (Figures 5D-F). These results suggest that ocln^−/−^ mice exhibited abnormal social recognition memory.

### Ocln-deficient mice display impaired novelty preference

**Figure 6.**
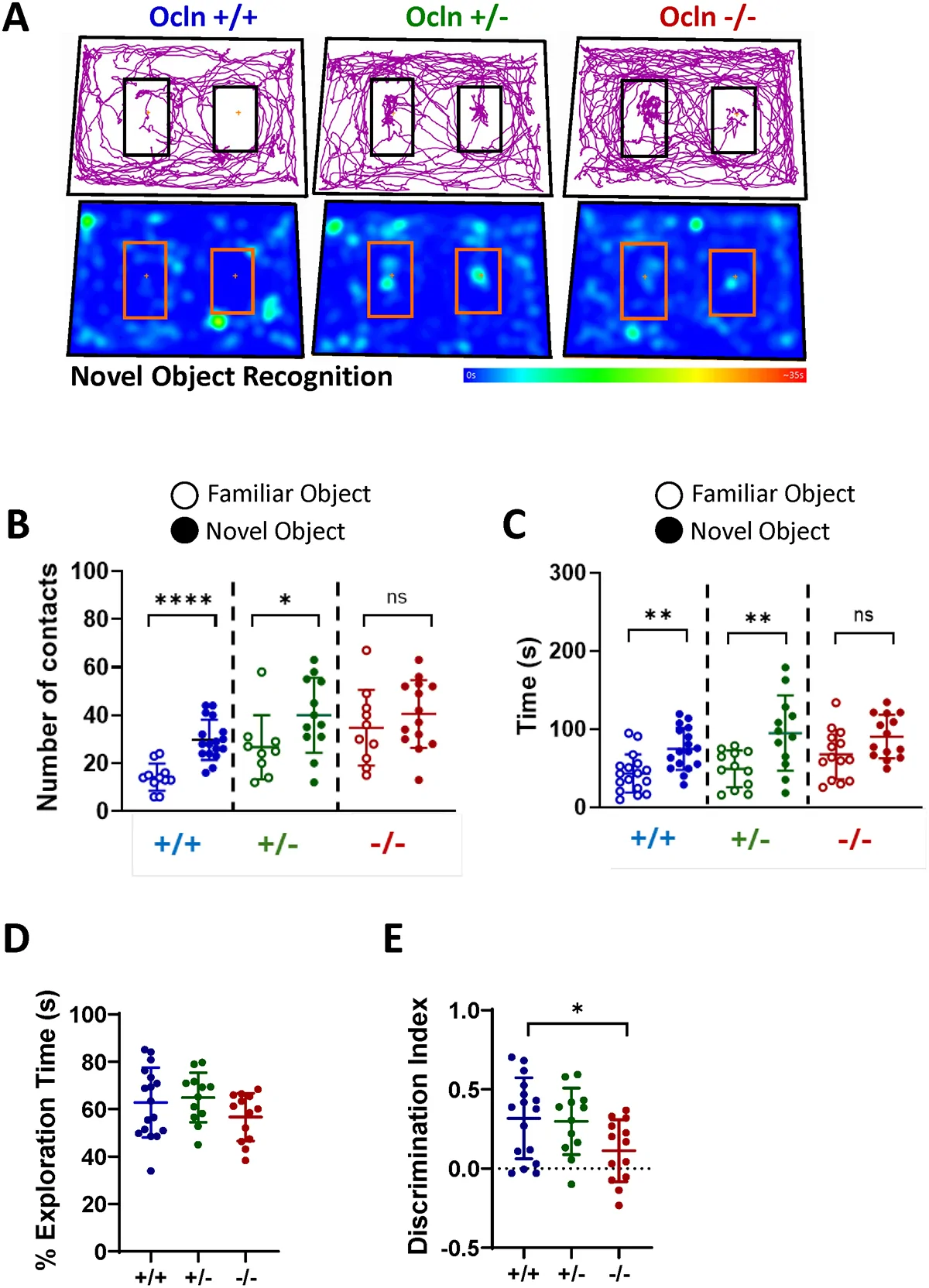
Ocln-deficient mice display impaired novelty preference. Novel object recognition test was recorded and analyzed using activity monitoring tracking software, ANY-maze Video tracking System. (A) Representative track pathway and heat map of ocln^+/+^, ocln^±^, and ocln^−/−^ mice. (B) the number of contacts with the familiar object and the novel object, (C) time spent with the familiar object and the novel object, (D) percentage of exploration time, and (E) the discrimination index. Individual data points are marked in blue (ocln^+/+^), green (ocln^±^), and red (ocln^−/−^) dots and circles. Graphs indicate the mean ± sd from three independent experiments. *p < 0.0449, *n* = 10–21 animals per group; unpair t-test. Data applies to both sexes.

We also examined cognitive functions by performing a novel object recognition test (Figure 6). Adult 12-week-old mice were placed in a box with two objects, a familiar object, and a novel object, which was never before seen by a mouse. Both wild-type and ocln^+^/^−^ mice demonstrated a significant preference for a novel object as demonstrated by the number of contacts and time spent with a novel object compared to the familiar object (Figures 6-C). In contrast, ocln^−^/^−^ mice failed to exhibit any preference for a novel object, indicating a potential deficit in novelty recognition. There were no differences in the total percentage of exploration time across genotypes (Figure 6D), suggesting that overall exploratory behavior was unaffected. Analysis of the discrimination index revealed a significant reduction in ocln^−^/^−^ mice compared to wild-type controls (Figure 6E). This decrease in discrimination index confirms that ocln^−^/^−^ mice were less able to distinguish between the familiar and novel objects, which is an indicator of a deficit in recognition memory. Wild-type mice revealed preferences for exploring the novel object as reflected by a positive discrimination index. Overall, these results indicate that ocln^−^/^−^ mice display impaired novelty preference, which may contribute to abnormal social recognition memory as observed in Figure 5.

### BBB dysregulation in ocln deficient mice

**Figure 7.**
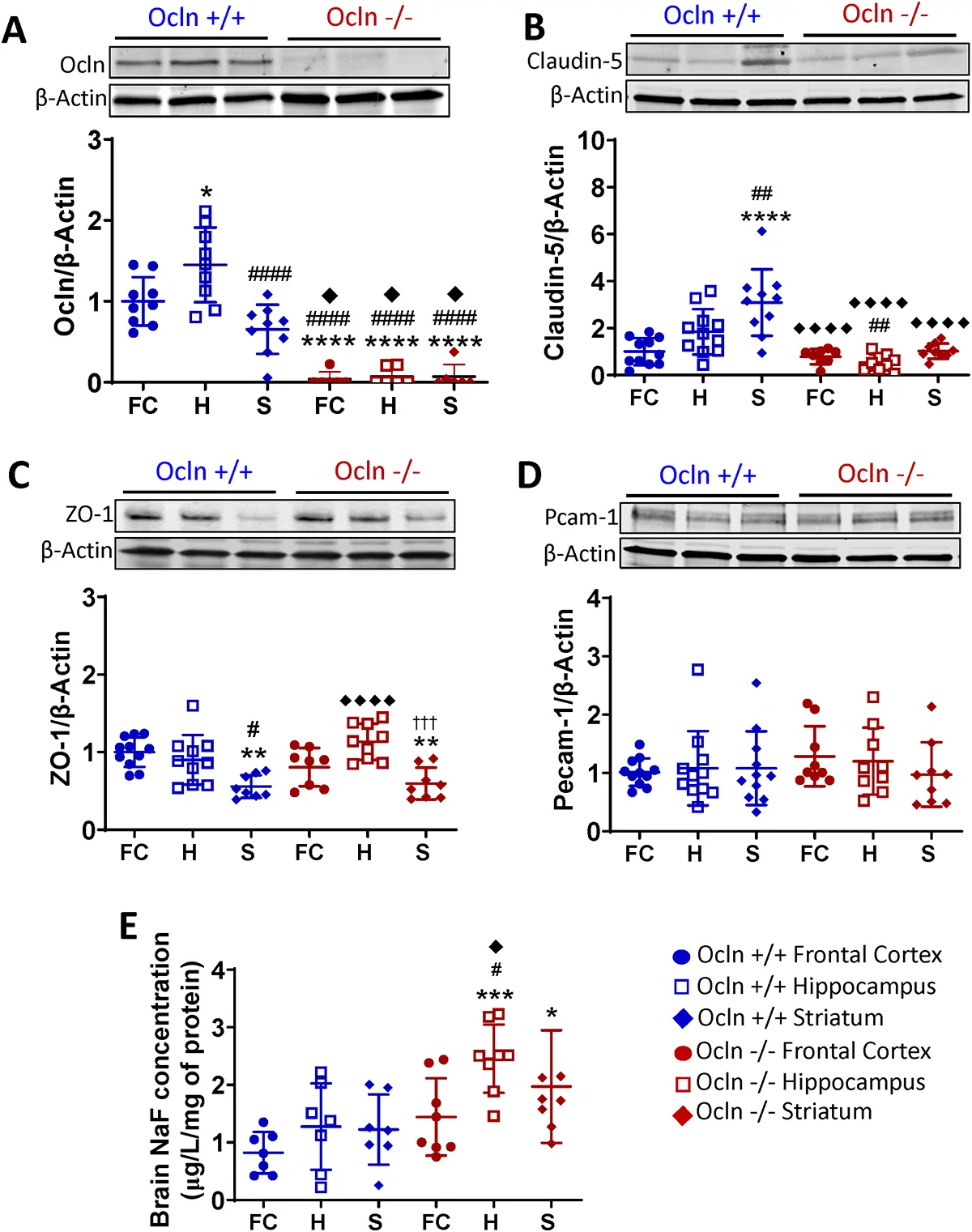
Compromised BBB integrity in ocln-deficient mice. Protein levels of (A) ocln, (B) claudin-5, (C) ZO-1 and (D), PECAM-1 in the frontal cortex (FC), hippocampus (H), and striatum (S) of ocln^−/−^ and ocln^+/+^ mice as analyzed by immunoblotting. (E) extravasation of NaF as the marker of BBB integrity in the frontal cortex (FC), hippocampus (H), and striatum (S) of ocln^−/−^ and ocln^+/+^ mice. Individual data points are marked in blue (ocln^+/+^) and red (ocln^−/−^) dots, squares, and diamonds. Graphs indicate the mean ± sd from three independent experiments. *vs. FC (ocln^+/+^) group at ****p < 0.0001, ***p = 0.0002, **p = 0.003, *p < 0.0449; ^#^vs. H (ocln^+/+^) group at ^####^*p* < 0.0001, ^##^*p* < 0.003, and ^#^*p* < 0.0449; ^♦^vs. S (ocln^+/+^) group at ^♦♦♦♦^*p* < 0.0001, and ^♦^*p* < 0.0449.; ^†^vs. H (ocln^−/−^) group at ^†††^*p* < 0.001, ^††^*p* < 0.01, and ^†^*p* < 0.0449, *n* = 7–11 animals per group; two-way ANOVA. Data applies to both sexes.

Damage to the BBB has been linked to several neurological disorders affecting the CNS.^6,8,9^ Although the role of ocln in regulating BBB integrity has been described,^23–25^ no studies have been performed comparing ocln levels between discrete brain regions. Mouse brains were dissected to isolate the frontal cortex (FC), hippocampus (H), and striatum (S), which were then probed for TJ proteins to assess BBB functionality. We observed significantly higher ocln levels in the hippocampus as compared to the frontal cortex or striatum in wild type (ocln^+/+^) mice (Figure 7A). In contrast, claudin-5 exhibited a significantly higher expression in the striatum compared to the frontal and hippocampus in these mice (Figure 7B). Interestingly, ocln^−/−^ mice exhibited notably lower levels of claudin-5 in the hippocampus and striatum compared to ocln^+/+^ mice (Figure 7B). Furthermore, ZO-1 expression demonstrated a significant decrease in the striatum in comparison to the frontal cortex or hippocampus, with no discernible differences between ocln^−/−^ and ocln^+/+^ mice (Figure 7C). Lastly, Platelet endothelial cell adhesion molecule (PECAM-1) expression levels were similar in all brain regions studied.

To functionally assess BBB permeability, sodium fluorescein was administered and quantified in brain tissues. Our results show a significantly higher NaF extravasation into the hippocampal brain parenchyma in ocln^−/−^ mice compared to ocln^+/+^ mice (Figure 7E), confirming that ocln protein deficiency enhances permeability to low-molecular-weight molecules in specific brain regions. On the other hand, no significant differences in total brain weight were observed between adult ocln^+/+^ and ocln^−/−^ mice (**Supplementary Figures S2A-B**), suggesting that occludin deletion does not affect gross brain volume.

## Discussion

The attention surrounding the role of blood-brain barrier (BBB) disruption and heightened permeability as risk factors for neurodevelopmental disorders (NDDs) has intensified in recent years. Serving as a dynamic boundary between the brain and periphery, the BBB has been implicated in various neurological diseases, including Alzheimer’s disease, traumatic brain injury (TBI), and epilepsy ^26,27^ as well as psychiatric disorders, including schizophrenia, autism spectrum disorder, and attention‐deficit hyperactivity disorder (ADHD) ^12,28^. Autism spectrum disorder is characterized by social and behavioral deficits, and a range of impairments, including deficits in learning,^4^ aggression ^5^ and hyperactivity.^29^ High levels of impulsivity, hyperactivity, and attention problems are also the major characteristics of ADHD.^30^

The barrier characteristics of the BBB are conferred by TJ proteins such as claudins, ocln, and zonula occludens, that limit the permeability of the BBB.^7^ Ocln is a multifunctional protein that can act as an NADH oxidase and modulate AMPK protein kinase activity.^16,18,20^ Despite its role as an important part of TJ complexes, the role of ocln in the development of neurological disorders has been poorly understood. In the present study, we found that adolescent ocln^−/−^ mice presented significant deficiencies in body weight, length, and neuroscore, when compared with ocln^±^ or ocln^+/+^ mice aged 3- and 6-week-old; however, these differences were less distinguished in 9- and 12-week-old mice (Figures 1, 2). While the mechanisms of impaired growth in ocln^−/−^ mice are not fully understood, they may be related to recently described metabolic functions of this protein, such as controlling glucose metabolism and involvement in redox-related processes. Indeed, ocln levels can alter glucose uptake, ATP content, and the expression levels of the glucose transporters GLUT-1 and GLUT-4.^20^ GLUT-1 facilitates glucose transport across plasma membranes, and it is the main transporter of glucose through the BBB.^31,32^ It has been reported that alterations in GLUT-1 levels can impair cerebral energy metabolism,^32^ fetal growth and development,^33^ and affect growth patterns ^34^ and nutritional status in children.^35^ In studies with children affected by Glucose Transporter 1 Deficiency Syndrome (GLUT1-DS), it has been reported that the body mass index z-score ranged from −1.5 to −1.9 in 30% of the children.^35^ Importantly, a recent study has suggested that GLUT1-DS is associated with growth impairment at birth, however, this impairment is decreased after puberty.^34^ A dynamic growth in young and adolescent mice was likely to potentiate these deficits, explaining the differences between growth deficits in 3- or 6-week-old ocln^−/−^ mice and the lack of differences between ocln^−/−^ and ocln^+/+^ mice at later ages. The impaired growth was also likely to contribute to abnormal footprint results in 3-week-old ocln^−/−^ mice (Figure 3). Because the growth differences between ocln^−/−^ and ocln^+/+^ mice were largely eliminated by week 12 of age, we performed open field, social novelty, and social interaction tests on 12-week-old ocln^−/−^ and ocln^+/+^ mice to assess if growth inhibition in adolescent mice translated to impaired social skills in adult mice.

The open field test is a widely used standard to evaluate motor activity, and emotional response, and it has been employed to study ADHD-type behavior in animals by evaluating levels of hyperactivity and impulsive behavior.^36,37^ Indeed, our results indicate that ocln^−/−^ mice present a higher level of hyperactivity when compared with wild-type mice (Figure 4). Hyperactivity and anxiety, which are behavioral characteristics of ADHD patients, are comorbidities often associated with autism.^38,39^ Importantly, the observed behavioral phenotypes, including comprising hyperactivity, attention deficits, and social impairments, are not fully specific to ASD or ADHD. These clinical symptoms are also present in other neuropsychiatric disorders, including schizophrenia. Furthermore, while our study assessed several behavioral domains such as social interaction, memory, and attention, we did not evaluate communication deficits (e.g., ultrasonic vocalizations in pups) or repetitive behaviors (e.g., marble burying), which are also core features of ASD. These limitations should be considered when interpreting the phenotype of ocln^−^/^−^ mice. It is also important to consider that the hyperactivity observed in ocln^−^/^−^ mice could be influenced by their reduced body size. Nevertheless, the increased activity was consistent across trials and accompanied by other behavioral and social deficits, supporting the notion that ocln deficiency resulted in a genuine neurobehavioral phenotype.

In line with our results, other studies have reported the potential involvement of TJ proteins in psychiatric diseases. For example, a correlation between ocln levels in people with bipolar disorder,^11^ and children with autism spectrum disorder ^12^ was reported. Additionally, reports indicated elevated levels of ocln in human postmortem brain tissue in individuals diagnosed with schizophrenia.^40^ Nevertheless, it should be noted that the initial report on the association between serum zonulin and ocln levels in patients with ADHD,^41^ has not been confirmed.^42^

Impaired social communication is a core symptom in autism spectrum disorder patients ^38^ and several studies have shown abnormal social behavior in a mouse model of this disease.^43,44^ Therefore, we next evaluated the social interaction and recognition tests in ocln^−/−^ mice. The results suggested that ocln^−/−^ mice exhibited impaired social interactions (Figure 5), which is a common symptom in autism spectrum disorder patients.^38^ Supporting these results, other studies have reported that mouse models lacking the autism spectrum disorder genes exhibited impairment in social novelty recognition,^45,46^ similar to those observed in our study in ocln^−/−^ mice.

Because a relationship between social novelty recognition and social novelty preference has been described in both autism spectrum disorder patients ^47^ and autism-like mouse models,^46,48^ we employed novel object recognition tests in ocln^−/−^ and control mice. In comparison to control mice, ocln^−/−^ mice exhibited hyperactivity and no apparent preference for a novel vs. a familiar object (Figure 6). Overall, the behavioral testing suggested that ocln^−/−^ mice present not only an impairment in memory but, more importantly, a deficit in attention and hyperactivity. These results agree with the report on a decrease in expression of ocln in the hippocampus of mice with impaired memory and learning ability.^49^

The findings of the present study could be influenced by the heterogeneity of brain regions in their content of ocln. Moreover, ocln deficiency could induce compensatory effects on other TJ proteins. Therefore, we evaluated ocln levels and their influence on the expression of other TJ proteins in the different regions of the brain. We selected the hippocampus, striatum, and frontal cortex for analysis based on their established involvement in memory, attention, emotional regulation, motor function, and social behavior, all domains that were altered in ocln-deficient mice. Our novel results indicated that ocln levels are significantly higher in the hippocampus when compared with the frontal cortex or the striatum. The potential implications of this funding may point to a higher susceptibility of the hippocampus to ocln deficiency as compared to other brain regions. Indeed, the mammalian hippocampus is a key brain structure for memory formation ^50^ and hippocampal impairment has been associated with several alterations, such as locomotor activity dysfunction, and psychosis;^51^ i.e., the functions that were largely affected in ocln^−/−^ mice. Nevertheless other brain regions, particularly the cerebellum, may also contribute to the observed behavioral phenotype. Traditionally associated with motor coordination, the cerebellum is increasingly recognized for its role in cognitive and affective processing, including social behavior and attention regulation.^52^ Although not evaluated in this study, the cerebellum remains an important target for future investigation to fully understand the regional impact of ocln deficiency on brain function and behavior.

Regarding the content of other TJ proteins in ocln deficiency, studies indicated lower levels of PECAM-1 protein expression in children and adults with autism spectrum disorder;^53,54^ however, our study did not find any influence of ocln on PECAM-1 levels. In addition, we did not observe any changes in ZO-1 expression levels of ocln^−/−^ mice. Interestingly, we found significantly lower expression levels of claudin-5 in the hippocampus and striatum of ocln^−/−^ mice when compared with ocln^+/+^ mice (Figure 7). These results have significant implications because studies have pointed to claudin-5 as a possible target in neurological and psychiatric disorders. Silencing of claudin-5 in mice was reported to lead to spontaneous recurrent seizures and neuroinflammation.^55^ Decreased claudin-5 levels have also been observed in the frontal cortex of individuals with schizophrenia,^28^ and abnormal claudin-5 levels have been implicated in depression.^56,57^ Claudin-5 knockdown in the nucleus acumens or downregulation of claudin-5 has been associated with worsened depression-like behaviors;^58^ however, higher levels of this protein were found in children with ADHD.^59^ Although no direct regulation between ocln and claudin-5 has been reported, several papers have shown that ocln and claudin-5 exhibit similar responses in different diseases.^12,60^ For example, downregulation of claudin-1, −5, ZO-1, and ocln was seen in brain capillaries of animals after inducing status epilepticus.^27^ These findings are important because modulation of claudin-5 levels may provide an additional potential explanation for the observed behavioral changes in ocln^−/−^ mice.

Claudin-5 is also the major regulator of BBB integrity;^12^ therefore, our studies included functional assessment of BBB permeability by the NaF extravasation method. We observed a significantly greater NaF extravasation into the hippocampal parenchyma in ocln^−^/^−^ mice compared to ocln^+^/^+^ controls (Figure 7), indicating BBB dysfunction. An association between increased BBB permeability and psychiatric disorders has been established in prior studies ^57,61^ and are consistent with our findings. While the present study assessed BBB permeability in adult mice, future investigations examining BBB integrity during earlier developmental periods are warranted to better understand the temporal progression of neurovascular and behavioral changes.

**Figure 8.**
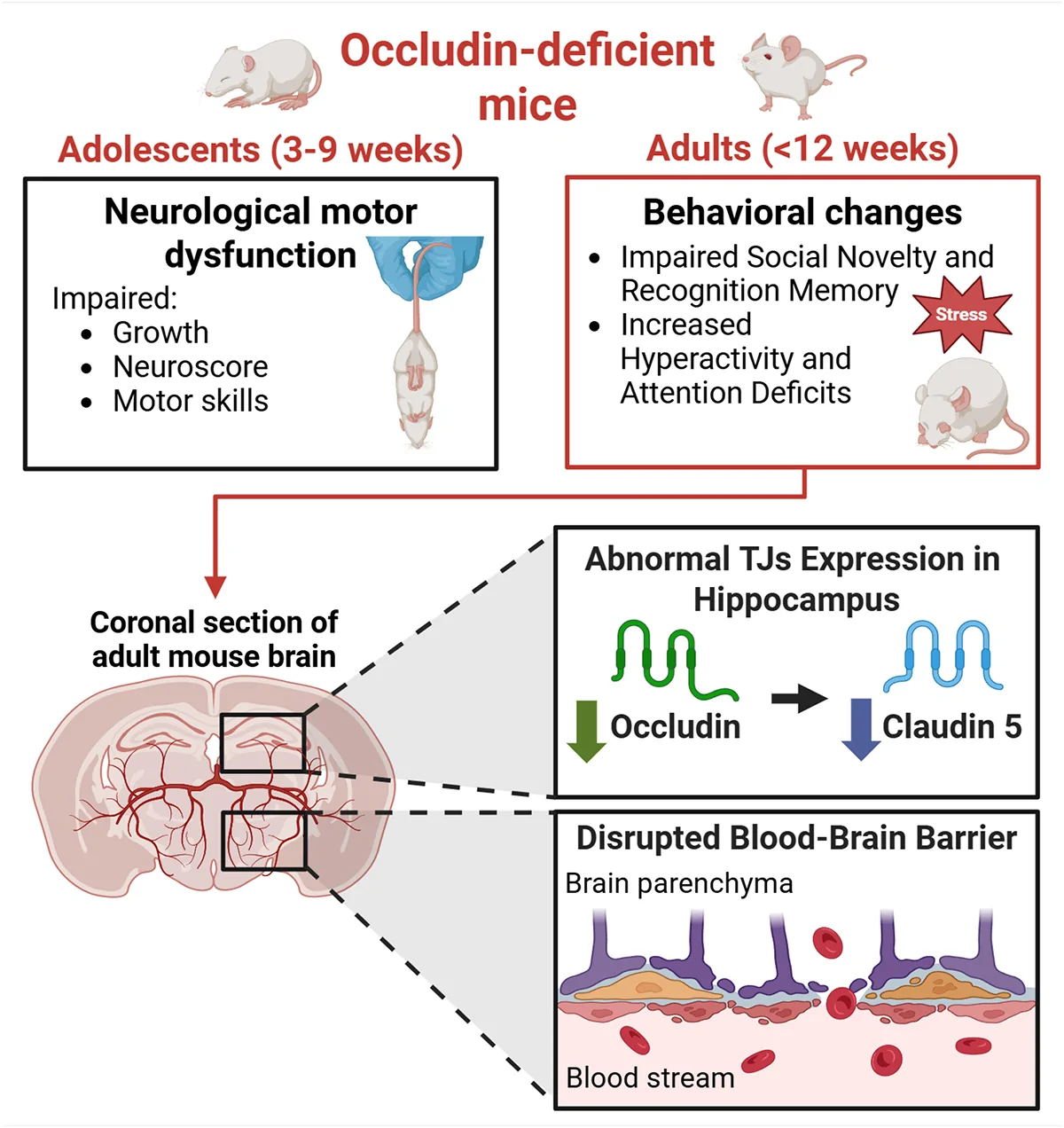
Proposed model of ocln-mediated neurodevelopmental disorders. Ocln-/- mice showed abnormal growth, motor issues, and increased hyperactivity, and social deficits. The hippocampus was most affected, with reduced levels of the tight junction protein claudin-5. These results suggest that BBB disruption may play a role in neurodevelopmental disorders. Ocln is identified as a potential target for future psychiatric research and therapies.

In conclusion, the present study reveals that ocln deficiency is associated with behavioral changes that are reminiscent of autism spectrum disorder, encompassing social and memory impairment, and attention-deficit/hyperactivity disorder. Furthermore, early, and adolescent growth as well as locomotor activity are impaired in ocln^−/−^ mice compared to those in wild-type mice. These observations contribute to a better understanding of the role of the ocln in neurodevelopmental disorders, particularly autism spectrum disorder and ADHD (Figure 8).

## Availability of data and materials

All source data supporting the findings of this manuscript are available from the corresponding authors upon request.

## Supplementary Material

Supplemental materials 10_17_2025rev-FINAL CLEAN.docx

Supplemental Table 1 10_17_2025rev.docx
