# Supplementary material for: Occludin deficiency is associated with developmental impairment, locomotor dysfunction, and social deficiencies
Source: Tissue Barriers. 2026 Mar 24;14(3):2647450. doi: 10.1080/21688370.2026.2647450 (PMC13540196; doi:10.1080/21688370.2026.2647450)
Supplement: Supplementary file 1 [file KTIB_A_2647450_SM9198.docx]

**Supp. Table 1: Detailed overview of statistical test and parameters used in this study**

| **Figure** | **Outcome Measure** | **Analysis Type** | **Factor Tested** | **F (value)** | **df (n, d)** | **p-value** | **Significant Comparisons** | **Interaction** |
| --- | --- | --- | --- | --- | --- | --- | --- | --- |
| Fig. 1 | Weight | Two-way ANOVA | Main: Genotype (+/+, +/-, -/-) | 48.72 | (2, 215) | <0.0001 | 3 weeks +/+ vs. -/- (*p*<0.0001), +/- vs. -/- (*p*<0.0001); 6 weeks +/+ vs. -/- (*p*<0.0001), +/- vs. -/- (*p*<0.0001); 9 weeks +/+ vs. +/- (*p*=0.0153), +/+ vs. -/- (*p*=0.0002); 12 weeks +/+ vs. +/- (*p*=0.0044), +/+ vs. -/- (*p*<0.0001), +/- vs. -/- (*p*=0.0259) |  |
|  |  |  | Main: Time | 256.6 | (3, 215) | <0.0001 |  |  |
|  |  |  | Genotype x Time Interaction | 0.8340 | (6, 215) | 0.5448 |  | No interaction |
|  | Length | Two-way ANOVA | Main: Genotype (+/+, +/-, -/-) | 27.51 | (2, 214) | <0.0001 | 3 weeks +/+ vs. -/- (*p*<0.0001), +/- vs. -/- (*p*<0.0001); 6 weeks +/+ vs. -/- (*p*<0.0001), +/- vs. -/- (*p*=0.0164), +/+ vs. +/- (*p*=0.0376); 9 weeks +/+ vs. -/- (*p*=0.0133); 12 weeks +/+ vs. +/- (*p*=0.0406), +/+ vs. -/- (*p*=0.0292) |  |
|  |  |  | Main: Time | 242.5 | (3, 214) | <0.0001 |  |  |
|  |  |  | Genotype x Time Interaction | 3.510 | (6, 214) | 0.0025 |  | Significant Interaction |
|  | NeuroScore | Two-way ANOVA | Main: Genotype (+/+, +/-, -/-) | 25.72 | (2, 214) | <0.0001 | 3 weeks +/+ vs. -/- (*p*<0.0001), +/- vs. -/- (*p*<0.0001), +/+ vs. +/- (*p*=0.0044); 6 weeks +/+ vs. -/- (*p*<0.0001), +/- vs. -/- (*p*<0.0001); 9 weeks +/+ vs. -/- (*p*=0.0284) |  |
|  |  |  | Main: Time | 62.53 | (3, 214) | <0.0001 |  |  |
|  |  |  | Genotype x Time Interaction | 11.00 | (6, 214) | <0.0001 |  | Significant Interaction |
| Fig. 2 | Weight (3 weeks) | Two-way ANOVA | Main: Genotype (+/+, +/-, -/-) | 31.00 | (2, 61) | 0.0001 | Male +/+ vs. Male -/- (*p*<0.0001), Male +/+vs. Female -/- (*p*<0.0001), Male +/- vs. Male -/- (*p*<0.0001), Male +/- vs. Female -/- (*p*=0.0049), Male -/- vs. Female +/+ (*p*<0.0001), Male -/- vs. Female +/- (*p*<0.0001), Female +/+ vs. Female -/- (*p*=0.0007), Female +/- vs. Female -/- (*p*=0.0043) |  |
|  |  |  | Main: Sex (Male, Femail) | 0.8660 | (1, 61) | 0.3557 |  |  |
|  |  |  | Groups x Sex Interaction | 0.8677 | (2, 61) | 0.4250 |  | No interaction |
|  | Length (3 weeks) | Two-way ANOVA | Main: Genotype (+/+, +/-, -/-) | 12.08 | (2, 60) | <0.0001 | Male +/+ vs. Male -/- (*p*=0.01), Male +/+ vs. Female -/- (*p*=0.0091), Male -/- vs. Female +/+ (*p*=0.0092), Male -/- vs. Female +/- (*p*=0.0010), Female +/- vs. Female -/- (*p*=0.0324) |  |
|  |  |  | Main: Sex (Male, Femail) | 2.710 | (1, 60) | 0.1050 |  |  |
|  |  |  | Groups x Sex Interaction | 0.2612 | (2, 60) | 0.7710 |  | No interaction |
|  | NeuroScore (3 weeks) | Two-way ANOVA | Main: Genotype (+/+, +/-, -/-) | 58.41 | (2, 57) | <0.0001 | Male +/+ vs. Male -/- (*p*<0.0001), Male +/+ vs. Female -/- (*p*=0.0004), Male +/- vs. Male -/- (*p*<0.0001), Male +/- vs. Female -/- (*p*=0.0100), Male -/- vs. Female +/- (*p*<0.0001), Male -/- vs. Female -/- (*p*<0.0001), Female +/+ vs. Female -/- (*p*=0.0016), Female +/- vs. Female -/- (*p*=0.0084) |  |
|  |  |  | Main: Sex (Male, Femail) | 15.23 | (1, 57) | 0.0003 |  |  |
|  |  |  | Groups x Sex Interaction | 3.695 | (2, 57) | <0.0001 |  | Significant Interaction |
|  | Weight (12 weeks) | Two-way ANOVA | Main: Genotype (+/+, +/-, -/-) | 9.542 | (2, 47) | 0.0003 | Male +/+ vs. Male -/- (*p*<0.0004), Male +/+ vs. Female +/+ (*p*<0.0001), Male +/+ vs. Female +/- (*p*<0.0001), Male +/+ vs. Female -/- (*p*<0.0001), Male +/- vs. Male -/- (*p*=0.0138), Male +/- vs. Female +/+ (*p*=0.0008), Male +/- vs. Female +/- (*p*<0.0001), Male +/- vs. Female -/- (*p*<0.0001) |  |
|  |  |  | Main: Sex (Male, Femail) | 66.20 | (1, 47) | <0.0001 |  |  |
|  |  |  | Groups x Sex Interaction | 2.014 | (2, 47) | 0.1448 |  | No interaction |
|  | Length (12 weeks) | Two-way ANOVA | Main: Genotype (+/+, +/-, -/-) | 8.792 | (2, 47) | 0.0006 | Male +/+ vs. Male +/- (*p*=0.0209), Male +/+ vs. Female +/- (*p*=0.0011), Male +/+ vs. Female -/- (*p*=0.0015) |  |
|  |  |  | Main: Sex (Male, Femail) | 4.142 | (1, 47) | 0.0475 |  |  |
|  |  |  | Groups x Sex Interaction | 0.3944 | (2, 47) | 0.6763 |  | No interaction |
|  | NeuroScore (12 weeks) | Two-way ANOVA | Main: Genotype (+/+, +/-, -/-) | 1.082 | (2, 48) | 0.3472 |  |  |
|  |  |  | Main: Sex (Male, Femail) | 3.277 | (1, 48) | 0.0765 |  |  |
|  |  |  | Groups x Sex Interaction | 1.031 | (2, 48) | 0.3643 |  | No interaction |
| Fig. 3 | Foodprint analysis | One-way ANOVA | Group: Sway Length | 7.241 | (2, 18) | 0.0049 | +/+ vs. -/- (*p*=0.02), +/- vs. -/- (*p*=0.0053) |  |
|  |  | One-way ANOVA | Group: Stance Length | 8.603 | (2, 20) | 0.0020 | +/+ vs. -/- (*p*=0.001) |  |
|  |  | One-way ANOVA | Group: Stride Length | 19.83 | (2,19) | <0.0001 | +/+ vs. -/- (*p*<0.0001), +/- vs. -/- (*p*=0.0005) |  |
| Fig. 4 | Open Field | One-way ANOVA | Group: Rotations of the animal's body | 3.161 | (2, 59) | 0.0496 | +/- vs. -/- (*p*=0.0477) |  |
|  |  | One-way ANOVA | Group: Distance travel | 7.208 | (2, 56) | 0.0016 | +/+ vs. -/- (*p*=0.0029), +/- vs. -/- (*p*=0.0053) |  |
|  |  | One-way ANOVA | Group: Average Speed | 7.222 | (2, 56) | 0.0016 | +/+ vs. -/- (*p*=0.0029), +/- vs. -/- (*p*=0.0054) |  |
|  |  | One-way ANOVA | Group: Number of entries to the peripheral zone | 6.212 | (2, 56) | 0.0037 | +/+ vs. -/- (*p*=0.0247), +/- vs. -/- (*p*=0.0035) |  |
|  |  | One-way ANOVA | Group: Time in the peripheral zone | 0.1053 | (2, 56) | 0.9002 |  |  |
|  |  | One-way ANOVA | Group: Number of entries to the inner zone | 4.610 | (2, 56) | 0.0140 | +/+ vs. -/- (*p*=0.0439), +/- vs. -/- (*p*=0.0148) |  |
|  |  | One-way ANOVA | Group: Time in the inner zone | 0.1053 | (2, 56) | 0.9002 |  |  |
| Fig. 5 | Social Interaction | Unpaired t-test | Group: Number of investigations +/+ | 5.559 | (14, 14) | 0.0028 | Stranger 1 +/+ vs. Empty +/+ (*p*<0.0001) |  |
|  |  | Unpaired t-test | Group: Number of investigations +/- | 2.759 | (12, 12) | 0.0915 | Stranger 1 +/- vs. Empty +/- (*p*=0.0004) |  |
|  |  | Unpaired t-test | Group: Number of investigations -/- | 11.34 | (12, 12) | 0.0002 | Stranger 1 -/- vs. Empty -/- (*p*<0.0001) |  |
|  |  | Unpaired t-test | Group: Time investigating +/+ | 1.536 | (14, 14) | 0.4318 | Stranger 1 +/+ vs. Empty +/+ (*p*<0.0001) |  |
|  |  | Unpaired t-test | Group: Time investigating +/- | 3.245 | (12, 12) | 0.0519 | Stranger 1 +/- vs. Empty +/- (*p*=0.0014) |  |
|  |  | Unpaired t-test | Group: Time investigating -/- | 4.167 | (12, 12) | 0.0198 | Stranger 1 -/- vs. Empty -/- (*p*<0.0001) |  |
|  | Social Recognition | Unpaired t-test | Group: Number of investigations +/+ | 1.553 | (15, 15) | 0.4036 | Stranger 1 +/+ vs. Stranger 2 +/+ (*p*=0017) |  |
|  |  | Unpaired t-test | Group: Number of investigations +/- | 1.987 | (13, 13) | 0.2290 | Stranger 1 +/- vs. Empty +/- (*p*=0.0002) |  |
|  |  | Unpaired t-test | Group: Number of investigations -/- | 1.141 | (12, 11) | 0.8341 |  |  |
|  |  | Unpaired t-test | Group: Time investigating +/+ | 1.679 | (15, 15) | 0.3262 | Stranger 1 +/+ vs. Stranger 2 +/+ (*p=0.0004*) |  |
|  |  | Unpaired t-test | Group: Time investigating +/- | 2.460 | (13, 13) | 0.1172 | Stranger 1 +/- vs. Empty +/- (*p*=0.0005) |  |
|  |  | Unpaired t-test | Group: Time investigating -/- | 1.348 | (12, 12) | 0.6133 |  |  |
| Fig. 6 | Novel Object Recognition | Unpaired t-test | Group: Number of contacts +/+ | 2.204 | (25, 92) | <0.0001 | Familiar Object +/+ vs. Novel Object +/+ (*p*<0.0001) |  |
|  |  | Unpaired t-test | Group: Number of contacts +/- | 2.101 | (18, 58) | 0.0496 | Familiar Object +/- vs. Novel Object +/- (*p*=0.0496) |  |
|  |  | Unpaired t-test | Group: Number of contacts -/- | 0.9104 | (18, 15) | 0.3745 |  |  |
|  |  | Unpaired t-test | Group: Time +/+ | 3.589 | (31, 71) | 0.0011 | Familiar Object +/+ vs. Novel Object +/+ (*p*=0.0011) |  |
|  |  | Unpaired t-test | Group: Time +/- | 2.964 | (15, 95) | 0.0092 | Familiar Object +/- vs. Novel Object +/- (*p*=0.0092) |  |
|  |  | Unpaired t-test | Group: Time -/- | 2.043 | (25, 64) | 0.06 |  |  |
|  |  | One-way ANOVA | Group: Exploration Time | 1.546 | (2,39) | 0.2259 |  |  |
|  |  | One-way ANOVA | Group: Discrimination Index | 3.418 | (2,38) | 0.0432 | +/+ vs. -/- (*p*=0.0500) |  |
| Fig. 7 | WB Occludin | Two-way ANOVA | Main: Genotype (+/+, +/-, -/-) | 118.8 | (1, 39) | <0.0001 | +/+ FC vs. -/- FC (*p*<0.0001), +/+ FC vs. +/+ H (*p*=0.0267), +/+ FC vs. -/- H (*p*<0.0001), +/+ FC vs. -/- S (*p*<0.0001), -/- FC vs. +/+ H (*p*<0.0001), -/- FC vs. +/+ S (*p*=0.0036), +/+ H vs. -/- H (*p*<0.0001), +/+ H vs. +/+ S (*p*<0.0001), +/+ H vs. -/- S (*p*<0.0001), -/- H vs. +/+ S (*p*=0.0068), +/+ S vs. -/- S (*p*=0.067) |  |
|  |  |  | Main: Brain Region (FC, H, S) | 6.703 | (2, 39) | 0.0031 |  |  |
|  |  |  | Groups x Brain Region Interaction | 6.599 | (2, 39) | 0.0034 |  | Significant Interaction |
|  | WB Claudin-5 | Two-way ANOVA | Main: Genotype (+/+, +/-, -/-) | 31.99 | (1, 52) | <0.0001 | +/+ FC vs. +/+ S (*p*<0.0001), -/- FC vs. +/+ S (*p*<0.0001), +/+ H vs. -/- H (*p*=0.0079), +/+ H vs. +/+ S (*p*=0.0097), -/- H vs. +/+ S (*p*<0.0001), +/ S vs. -/- S (*p*<0.0001) |  |
|  |  |  | Main: Brain Region (FC, H, S) | 10.76 | (2, 52) | 0.0001 |  |  |
|  |  |  | Genotype x Brain Region Interaction | 6.367 | (2, 52) | 0.0034 |  | Significant Interaction |
|  | WB ZO-1 | Two-way ANOVA | Main: Genotype (+/+, +/-, -/-) | 0.1691 | (1, 48) | 0.6827 | +/+ FC vs. +/+ S (*p*=0.0021), -/- FC vs. -/- S (*p*=0.0061), -/- H vs. -/- S (*p*=0.0343), +/+ S vs. -/- H (*p*<0.0001), -/- H vs. -/- S (*p*=0.0002) |  |
|  |  |  | Main: Brain Region (FC, H, S) | 16.48 | (2, 48) | <0.0001 |  |  |
|  |  |  | Genotype x Brain Region Interaction | 3.944 | (2, 48) | 0.0260 |  | Significant Interaction |
|  | WB Pecam-1 | Two-way ANOVA | Main: Genotype (+/+, +/-, -/-) | 1.400 | (1, 53) | 0.2420 |  |  |
|  |  |  | Main: Brain Region (FC, H, S) | 0.3334 | (2, 53) | 0.7180 |  |  |
|  |  |  | Genotype x Brain Region Interaction | 1.021 | (2, 53) | 0.3671 |  | No interaction |
|  | NaF | Two-way ANOVA | Main: Genotype (+/+, +/-, -/-) | 16.82 | (1,39) | 0.0002 | FC^+/+^ vs. H^-/-^ (*p*=0.0007), FC^+/+^ vs. S^-/-^ (*p*=0.0301), H^+/+^ vs. H^-/-^ (*p*=0.0239), S^+/+^ vs. H^-/-^ (*p*=0.0168) |  |
|  |  |  | Main: Brain Region (FC, H, S) | 4.280 | (2, 39) | 0.0209 |  |  |
|  |  |  | Genotype x Brain Region Interaction | 0.6735 | (2, 39) | 0.5158 |  | No interaction |
| Supp. Fig. 1 | Open Field | Unpaired t-test | Group: Total Distance | 2.227 | (9, 5) | 0.3915 |  |  |
|  |  | Unpaired t-test | Group: Average Speed | 2.232 | (9, 5) | 0.3900 |  |  |
|  |  | Unpaired t-test | Group: Number of entries to the peripheral zone | 1.1917 | (7, 7) | 0.4099 |  |  |
|  |  | Unpaired t-test | Group: Number of entries to the inner zone | 4.017 | (9, 5) | 0.1400 |  |  |
| Supp. Fig. 2 | Brain Weight | Unpaired t-test | Group: Brain Weight | 1.062 | (5, 5) | 0.9493 |  |  |
