# Supplementary material for: Occludin deficiency is associated with developmental impairment, locomotor dysfunction, and social deficiencies
Source: Tissue Barriers. 2026 Mar 24;14(3):2647450. doi: 10.1080/21688370.2026.2647450 (PMC13540196; doi:10.1080/21688370.2026.2647450)
Supplement: Supplementary file 2 [file KTIB_A_2647450_SM9199.docx]

**Occludin deficiency is associated with developmental impairment, locomotor dysfunction, and social deficiencies**

Silvia Torices^1*^, Nikolai Fattakhov^1^, Oandy Naranjo^1^, Destiny Daniels^1^, Sierra Simon^1^, Leah Daire^1^, Luisa Mendoza^1^, Marta Kopanska^2^ and Michal Toborek^1,3*^

**Supplementary Materials**

**Figure Legends**

**Supp. Figure 1. Impact of gender on open filed test results in ocln deficient mice.** Related to Figure 4. Open field test was recorded and analyzed using activity monitoring tracking software, ANY-maze Video Tracking System. **(A)** The total distance traveled, **(B)** average speed, **(C)** number of entries to the peripheral zone, and **(D)** time in the peripheral zone was compared between female and male ocln deficient mice. Individual data points are marked in red (ocln^-/-^). Graphs indicate the mean ± SD from three independent experiments, n=6-10 animals per group. Data applies to separate sexes.

**Supp. Figure 2.** **Comparison of brain weight between wild-type and occludin deficient mice.** **(A)** Representative images. **(B)** Brain weight was measured in wild-type (+/+) and occludin deficient (-/-) mice. No significant differences were observed between the groups. Individual data points are marked in blue (ocln+/+), and red (ocln-/-) dots. Graphs indicate the mean ± SD from three independent experiments, n=6 animals per group; unpair t-test.
